# Nurses’ Perception Regarding the Quality of Communication between Nurses and Physicians in Emergency Departments in Saudi Arabia: A Cross Sectional Study

**DOI:** 10.3390/healthcare11050645

**Published:** 2023-02-22

**Authors:** Nawal Daheshi, Sameer A. Alkubati, Hazel Villagracia, Eddieson Pasay-an, Ghadeer Alharbi, Farhan Alshammari, Norah Madkhali, Bushra Alshammari

**Affiliations:** 1 Jazan Academic Affairs and Training Administration, Jazan 85539, Saudi Arabia; 2 Medical Surgical Nursing Department, College of Nursing, University of Hail, Hail 2440, Saudi Arabia; 3 Department of Nursing, Faculty of Medicine and Health Sciences, Hodeida University, Hodeida P.O. Box 3114, Yemen; 4 Maternal and Child Nursing Department, College of Nursing, University of Hail, Hail 2440, Saudi Arabia; 5 School of Nursing and Midwifery, Queen’s University Belfast, Belfast BT7 1NN, UK; 6 Department of Pharmaceutics, College of Pharmacy, University of Hail, Hail 2440, Saudi Arabia; 7 Department of Nursing, College of Nursing, Jazan University, Jazan 45142, Saudi Arabia

**Keywords:** quality, communication, nurses, physician, emergency department, Saudi Arabia

## Abstract

**Background:** One of the defining characteristics of safe and highly reliable patient care is effective team communication. It is becoming increasingly crucial to improve communication among healthcare team members since social and medical conditions change quickly. **Main aim:** The present study seeks to assess nurses’ perception of the quality of communications between physicians and nurses and associated factors in the emergency departments of selected government hospitals in Saudi Arabia. **Methods:** A cross-sectional study was carried out in five hospitals in Jazan and three hospitals in Hail City, Saudi Arabia, on a convenience sample of 250 nurses total using self-administered questionnaires. Independent sample t-tests and one-way ANOVA were used for the data analysis. Ethical considerations were adhered to throughout the conduct of the study. **Results:** The mean score of all domains of nurses’ perceptions of the quality of nurse–physician communication in emergency departments was 60.14 out of 90. The highest mean score was observed in the openness subdomain, followed closely by relevance and satisfaction, with mean percentages of 71.65% and 71.60%, respectively. Age, level of education, years of experience, and job position had significant positive correlations with nurses’ perceptions of the quality of nurse–physician communication. (*p* = 0.002, 0.016, 0.022, and 0.020, respectively). Post hoc tests showed that nurses older than 30, those with diplomas, those with more than 10 years’ experience, and those in supervisory positions had more positive perceptions of the quality of nurse–physician communication. On the other hand, there was no significant difference in the mean scores of quality of nurse–physician communication with regard to participants’ sex, marital status, nationality, and working hours (*p* > 0.05). Multiple linear regression showed that none of the independent factors affected the nurses’ perceptions of the quality of nurse–physician communication in emergency departments (*p* > 0.05). **Conclusions:** Overall, the quality of communication between nurses and physicians was not satisfactory. Future research should be meticulously planned, using validated outcome measures, that will capture and reflect the goals of communication among healthcare teams.

## 1. Introduction

Hospitals around the world are placing an increasing emphasis on the standard of patient care and safety [1]. The ability of a healthcare team to communicate effectively is directly tied to the ability to treat patients in ways that are both safe and reliable [2]. In view of the current social and medical turmoil, it is becoming more and more crucial for healthcare team members to enhance their ability to communicate with one another [1]. Weller et al. found that hospitals depend heavily on nurses and physicians, two of the most vital professions in the healthcare sector [2]. In order to give patients the best care possible, they must be able to communicate effectively when carrying out their clinical duties [3]. Effective nurse–physician communication depends on sending the right information to a doctor while making sure that the recipient understands what is being conveyed [4]. Communication in the emergency department is particularly challenging due to the nature and demands of both patients and healthcare workers. On an interprofessional level, collaborations between nurses and physicians lack the foundational relationships that could affect the clinical experience [5], while contextual factors such as an increased number of patients and lower hospital staff numbers are exacerbating factors [6]. According to Hailu et al. [7], there was an association between the quality of communication and a wide variety of variables, including age, particular beliefs and expectations, educational background, and credentials [7]. Lack of time during medical encounters can become a barrier to the growth of a caring relationship because it calls for highly effective communication [8]. Other hindrances to successful communication include the workplace environment and a lack of experience among the healthcare team in different specialties [9]. There is limited research in Saudi Arabia on how to evaluate the quality of communication among healthcare teams, but it has been reported that ineffective communication can lead to two thirds of sentinel events in healthcare [10]. Medical sentinel events can happen at any time of day and having open communication among healthcare teams is a vital component of patient safety. A systematic review study of collaboration among nurses and physicians is highly recommended to maintain collegial and positive relationships among coworkers [11]. As the frontline workers in healthcare, nurses and physicians each have specific duties and responsibilities, but they are expected to deal with one another for the benefit of the patients; patient care plans that will make both nurses and physicians aware of what is expected will improve communication between them [12]. Streeton et al. [13] emphasized the importance of good interaction for effective communication amidst difficulties in the emergency department. One of the most significant factors directly linked to medical errors is a lack of collaboration and communication [14]. The identification of different strategies that could lead to effective communication among healthcare teams helps to improve patient outcomes [8].

Studies evaluating nurses and physicians in the emergency department in terms of communication are still lacking; although in this current study, the researchers acknowledge the importance of having a thorough understanding of the quality of communication between emergency nurses and physicians to improve the standards of nursing care provided to patients and to minimize the number of medical errors. Several interventions were used to increase nurse–physician communication. Multistructured work shift evaluation examines team training, tools/checklists, situation-background assessment and recommendation (SBAR) documentation templates, and communication [8]. Nurses’ and residents’ understanding of the day’s goals of care significantly improved when the daily goals form was utilized and implemented, thus, intensive care and length of stay has been reduced [8]. Since the present study focuses on the quality of communication between nurses and physicians in emergency departments in Saudi Arabia, it may be of use to authorities in other Middle Eastern countries to evaluate the communication strategies used by their healthcare professionals, both with one another and with patients. To enhance communication and ensure effective information transmission, a variety of communication strategies can also be used in the healthcare industry. The patient experience can be significantly enhanced by improving communication between all members of the care team and by communicating with patients more efficiently [15]. The present study specifically sought to (1) explore nurses’ perceptions of the quality of communication between physicians and nurses in several Saudi emergency departments and (2) identify factors that associated with the quality of communication between physicians and nurses.

## 2. Materials and Methods

### 2.1. Design and Setting

A cross-sectional design was used to determine nurses’ perceptions of the quality of communication between nurses and physicians in the emergency departments of eight government hospitals in Jazan and Hail Provinces in the Kingdom of Saudi Arabia. In Jazan, five hospitals were studied: (1) Sabia General Hospital, (2) Beesh Hospital, (3) King Fahad Hospital, (4) Abu Arish Ministry of Higher Education General Hospital, and (5) Prince Mohammed bin Nasser Hospital. In Hail City, three hospitals were part of the study: (1) King Salman Hospital, (2) King Khaled Hospital, and (3) Hail General Hospital. Multicenter research has its own advantages, compared to single-center studies, as it offers a quicker recruitment of the necessary number of participants, larger representative sample size with clearer and convincing results as well as allowing for a better basis for the subsequent generalization of its findings [16].

### 2.2. Sample

The study population consisted of staff nurses working in emergency departments at public hospitals in Jazan and Hail. The Raosoft sample size calculator [11] was used to compute a sample size that would deliver a 95% confidence interval: α = 0.05, and *N* = 582, demonstrating that 232 nurses were required. The sample was increased to involve 250 nurses working in the seven hospitals’ emergency departments between January and June 2022. Assistant nurses and training nurses were excluded, as were nurses working in other departments. A convenience sample was used to collect data in this study.

### 2.3. Instrument

The questionnaire consisted of two parts (see Supplementary S1). Part I concerned participants’ demographic characteristics: age, sex, educational level, job position, marital status, working hours per day, nationality, and years of experience. Part II was a standardized questionnaire adopted from Schmidt and Svarstad (2002) to assess the quality of communication between nurses and physicians [17]. This questionnaire contained 18 items across four dimensions: openness (4 items), relevance and satisfaction (8 items), mutual understanding (2 items), and frustration with interaction (4 items). All items were measured on a five-point Likert scale ranging from 1 = none, not at all, or very difficult to 5 = a lot, always, extremely, or very easy. Thus, the total score of the questionnaire ranged from 18 to 90. The questionnaire underwent validity and pilot testing for its reliability, with a Cronbach’s alpha of 0.805.

### 2.4. Data Collection

After completion of the pilot study, the researchers reached out to the head of nurses in the intended hospitals to encourage nurses to participate. The researchers then approached nurses and invited them to complete the questionnaire. Further, the questionnaires and informed consent were distributed during nurses’ break time after outlining the study’s goals. Further, questionnaires were distributed to participants during their break time. During the data collection, an explanation of the study tool was offered to participants to allay any concerns about the study questions. Nurses were asked to return the completed questionnaire to the head nurses. Data were collected between January and June 2022.

### 2.5. Ethical Considerations

Ethical and administrative considerations were dealt with before data collection began. Ethical approval was obtained from Jazan health ethics committee (No: 2226/2022) and the IRB of Hail health cluster in Hail City (No: 10/2022). Administrative approval was also obtained from the selected hospitals. The confidentiality of the information provided was assured since participants’ names were not included in the questionnaires. The study was anonymous and an explanation of the research process was provided to participants by the researchers. The participants were assured that their participation in the study was voluntary and that they had the right to withdraw at any stage of the research without having to offer a reason. In addition, consent forms were obtained from all participants.

### 2.6. Data Analysis

SPSS v. 24 (IBM, Chicago, IL, USA) was used to analyze the data. After data collection was completed, the data were coded and transferred to SPSS. Descriptive statistics such as frequencies, percentages, means, and standard deviations were used to describe participants’ characteristics. The normality of distribution was assessed using the Kolmogorov–Smirnov test. The results indicated that *p* value more than 0.05, that means the data were normally distributed. Consequently, parametric statistics were used in this study. Independent sample t-tests and one-way ANOVA were used to determine the factors affecting nurses’ perceptions of the quality of nurse–physician communication in emergency departments. Independent sample *t*-tests were used to determine differences in the mean scores of questionnaire items with two categories, such as sex, nationality (Saudi or other), and working hours (more or fewer than eight hours a day), while one-way ANOVA was used to determine differences in the mean scores of questionnaire items with categorical independent variables that involved more than two categories: age, level of education, experience in years, job position, and marital status. Factors significantly associated with nurses’ perceptions were further analyzed using multiple linear regression to determine the independent predictors of nurses’ perceptions of the quality of nurse–physician communication in emergency departments. Statistical significance was set at *p* ˂ 0.05.

## 3. Results

### 3.1. Sociodemographic Characteristics of Participants

Table 1 illustrates the sociodemographic characteristics of participants (*N* = 250). The mean age of participants was 32.65 years; 78.4% were female. Approximately two thirds and three quarters of participants were married and from Saudi Arabia (66.4% and 78.0%, respectively). In addition, mean years of experience in emergency departments was 9.28 ± 5.67. The majority of participants worked eight or fewer hours a day and worked as bedside nurses (65.6% and 70.4%, respectively). Finally, more than half (58.8%) of participants had a bachelor’s degree.

### 3.2. Nurses’ Perceptions of Quality of Nurse–Physician Communication

Table 2 shows nurses’ perceptions of the quality of nurse–physician communication in Saudi emergency departments. The total mean score was 60.14 out of 90 (range: 26–85), with the highest mean scores observed in “Difficult or easy to ask physicians for advice” and “Feeling respected after interaction with physicians”, with mean scores of 3.72 and 3.70, respectively, followed by the item “Difficult or easy to talk openly with physicians”, with a mean score of 3.64. The lowest score was observed in the item “Feeling frustrated after interaction with physicians” with a mean score 2.66.

### 3.3. Subscale Scores of Nurses’ Perceptions of Quality of Nurse–Physician Communications in Emergency Departments

The highest mean score was observed in the subdomain “openness subdomain”, fol-lowed closely by “Relevance and Satisfaction”, with mean percentages of 71.65% and 71.60%, respectively. The lowest mean score was observed in the subdomain “Frustration with Interaction” subdomain, with a mean percentage of 55.80% (see Table 3).

### 3.4. Factors Affecting Nurses’ Perceptions of Quality of Nurse–Physician Communication in Emergency Departments

Table 4 illustrates the factors affecting nurses’ perceptions of the quality of nurse–physician communication in emergency departments. Age, level of education, years of experience in emergency departments, and job position had a significant positive relationship with those perceptions (*p* = 0.002, 0.016, 0.022, and 0.020, respectively). Post hoc tests showed that nurses aged 30 or older, those with more than 10 years’ experience in emergency departments, and those in supervisory positions had more positive perceptions of the quality of nurse–physician communication. On the other hand, there was no significant difference in the mean scores of the quality of nurse–physician communication with regard to participant sex, marital status, nationality, or working hours (*p* > 0.05; see Table 4).

### 3.5. Multiple Linear Regression of Independent Factors of Nurses’ Perceptions of the Quality of Nurse–Physician Communication

Multiple linear regression showed that none of the independent factors affected the nurses’ perceptions of the quality of nurse–physician communication in the emergency departments (*p* > 0.05) (see Table 5).

## 4. Discussion

The present study examines nurses’ perceptions of the quality of nurse–physician communication in emergency departments in Saudi Arabia. Those perceptions were observed to be high in the openness domain, which means that nurses are comfortable with the physicians and achieve a depth of knowledge via the conversation. Consequently, the nurses feel that their goals are met and that dialogue is successful, as they can talk with physicians easily and openly; this may be because the nurses are familiar with the physicians. Studies in Ethiopia [18,19] yielded similar results. Physicians, however, were found to be more forthcoming with information than nurses in the literature. In Kim et al.’s study [20], physicians were found to have a more positive assessment of the openness of communication (73%) than nurses (32%). In another study, at least 70% of physicians believed they had good communication with nurses, but only 35–67% of nurses felt the same way [21]. One possible explanation for these results may be that physicians place less value on nurses’ input because they view their own work as more important.

The relevance of and satisfaction with communication between physicians and nurses were observed, which indicate that nurses are pleased with the communication that they have with the physicians and that physicians recognize the importance of discussions with nurses. According to House and colleagues [22], communication needs to be complete (containing all pertinent information), clear (conveyed in a way that is clearly recognized), brief (presented in a manner that is concise), and timely (accessible within a suitable time frame for effective clinical actions) to be effective [22]. Mutual understanding was the third highest score (60.10), placing it in the average to good category. Research by Aghamolaei and associates is supported by this parameter [23]. Having common ground may be the most important aspect of effective communication [24]. Therefore, hospital administrations should make efforts to improve this vital aspect of communication, de-spite the fact that the score in this area is not so low as to be a matter of obvious and im-mediate concern.

It should be noted that frustration with interactions had the lowest mean score, which implies that this obstacle to communication still exists in Saudi emergency rooms. The inability of physicians and nurses to effectively communicate with one another can have negative consequences for patients, including increased hospital lengths of stay, patient readmissions, and avoidable morbidity and mortality [25]. The necessity to find solutions to difficult communication issues leads to unwelcome emotional outbursts, which, in turn, may cause new issues to arise or make existing ones worse [26]. The findings of the present study highlight the importance of nurse–physician communication and the factors that can help strengthen it, thus improving the quality of care provided to patients and the outcomes they experience in emergency departments. Nurses’ input to physicians should be encouraged to maintain patient safety by fostering an environment conducive to open communication between healthcare workers through strong management. In addition, the findings of the present study suggest training nurses and physicians together to prevent any underestimation of the contributions of each specialty. This research can be used to build communication recommendations between nurses and physicians.

Age (30 or older), level of education (diploma), years of experience (more than 10 years), and job position (supervisor) all had significant positive correlations with nurses’ perceptions of the quality of nurse–physician communication, which indicates that both nurses’ demographics and nurses’ perceptions point in the same direction. All of these factors indicate a greater amount of experience gained over time. As stated in Nikandish and associates’ study, improved nurse–physician communication may result from nurses acquiring more information and training [27], which older nurses and nurses with years of experience have. Furthermore, according to the findings of Kang and colleagues [28], the clinical outcomes of patients were directly influenced by a variety of professionals’ levels of education as well as their ability to collaborate. It is possible that this occurs because the nurse and the physician are having trouble communicating with one another due to language barriers [29]. The key to effective communication in the emergency room may lie in the nurses’ professional training and awareness of language and cultural barriers that has developed over the course of their careers. The findings of the present study are consistent with those from previous research conducted on patterns of nurse–physician communication in patient care in the United States [3] and on nurse–physician relationships in Rwanda [30]. In contrast to the findings presented here, a study in Turkey [31] and research in Ethiopia among nurses [19] found that older nurses have the lowest levels of communication skills. Hailu et al. [7] also found a negative correlation between participants’ ages and levels of communication [7]. The fact that most people in the present study sample were under 30 years old may account for some of this disparity; it could also be attributed to differences in study design and sample size. In order to achieve the best possible outcomes for patients, enhancing communication between nurses and physicians requires several tactics. The development of tools that can accurately capture and reflect the influence of effective communication will make it easier to conduct accurate evaluations of treatments linked to communication and will reduce the amount of variation in outcome measures. Additionally, the feasibility of the intervention needs to be taken into account.

On the other hand, there was no significant difference in the mean scores of the quality of nurse–physician communication with regard to participant sex, marital status, nationality, or working hours, which means that there is no discernible difference in the quality of communication between physicians and nurses related to those characteristics. These results are in line with those reported in earlier research on nurse–physician communication in Iran and Ethiopia. According to Degavi, there were no discernible variations in nurse–physician communication between younger and older nurses or related to education and length of service [32]. By contrast, Teshnizi and colleagues found that female nurses consider physician–nurse communication to be much better than male nurses, based on years of experience in emergency departments [26]. Previous research has identified potential impediments to effective nurse–physician communication such as inadequate communication skills, misunderstanding of roles, perceived inequalities in the positions of nurses and physicians, variations in educational level, role expectations, and sex challenges [33]. While the same causes may be responsible for nurses’ perceptions of nurse–physician communication in the present study, more research is required to confirm their applicability to the Saudi nursing context.

The demographics of the participants in this study (e.g., age, level of education, experience, and job position) did not affect the nurses’ perceptions of the quality of nurse–physician communication in emergency departments. This indicates that nurses’ perceptions of the quality of nurse–physician communication are consistent across demographics. It is possible that this is due to the fact that these nurses are appreciated by the physicians, who solicit their input and invite them to the bedside while they are seeing a patient. Instead of just issuing directives to nurses, they would demonstrate a problem-solving strategy that emphasized the importance of teamwork [29]. By working together toward the same patient care goals, the aforementioned strategies can help nurses and physicians overcome power differential concerns [26] that might hinder successful nurse–physician communication. Similar results were observed by Pakpour et al. [34], who found no link between nurses’ age and their perspective on nurse–physician communication. Consistent with the findings of some earlier research, additional studies have found no connection between nurses’ perceptions of the quality of nurse–physician communication and demographic variables such as years of experience, shift type, marital status, or number of children [34,35]. However, this finding runs counter to what was reported in other research on the characteristics of nurse–physician communication in Ethiopia [7,19] and on the patterns of nurse–doctor relationships in Rwanda [30]. As specified in Jemal et al.’s study, the degree of nurse–physician communication declined as the ages of nurses and physicians increased [19]. In addition, according to Hailu et al.’s study, nurses with a higher education level had a higher mean score in nurse–physician communication satisfaction [7], which can be explained by the fact that nurses’ role expectations tend to rise along with their level of education. This disparity could be the result of differences in study environments and the composition of the samples. This finding can be of practical use to hospital administrators. There needs to be continued work to improve crossdepartmental communication between the emergency room and the rest of the hospital, as many issues that surface there are better understood as hospital-wide problems. It is unjust and unsustainable for hospital administrations to try to ignore or deflect attention from the emergency department because of the fundamental role it will play in the future of any healthcare institution. When it comes to the overall success and image of a hospital, the emergency department’s level of critical thinking and decision making matters a great deal.

### Limitations

As with any research, the present study has certain limitations. The findings were obtained from nurses’ insights into the quality of communication between nurses and physicians, and it would be valuable to explore the views of physicians in future studies. Culturally based misunderstandings could influence levels of communication, an aspect that was not explored. Messages can be understood differently in different cultures. The Saudi Arabian health system is heavily staffed by non-Saudi health professionals, especially physicians [36], while the majority of nurses are Saudi. It has been reported that females nurses have lower communication and teamwork skills than male nurses [37]. As the participants in this study were predominantly female, communication might be influenced by traditional female socialization in Saudi culture, which should be considered in future studies. A convenience sample was used in this study; this raises the possibility of sampling bias and means that the findings may not be generalizable to other settings.

## 5. Conclusions

The age, level of education, years of experience, and job position had significant positive correlations with nurses’ perceived of quality of nurse–physician communication. Conversely, there was no significant difference in the mean scores of the quality of nurse–physician communication with regard to participants’ sex, marital status, nationality, and working hours. None of the independent factors affected the nurses’ perceptions of the quality of nurse–physician communication in emergency departments. Overall, the quality of communication between nurses and physicians was not satisfactory. Future research should be meticulously planned, using validated outcome measures that will capture and reflect the goals of communication among healthcare teams.

## Figures and Tables

**Table 1:** Sociodemographic characteristics of participants (*N* = 250).

Characteristics	n	%
Sex		
Male	54	21.6
Female	196	78.4
Age		
<30	77	30.8
30–35	103	41.2
≥36	70	28.0
	Mean ± SD: 32.65 ± 5.59	
Education		
Diploma	66	26.4
Bachelor	147	58.8
Master	37	14.8
Experience in ED (years)		
1–5 years	77	30.8
6–10	72	28.8
>10	101	40.4
	Mean ± SD: 9.28 ± 5.67	
Job position		
Bedside nurse	176	70.4
Head nurse	40	16.0
Nursing supervisor	34	13.6
Marital status		
Single	75	30.0
Married	166	66.4
Divorced	9	3.6
Nationality		
Saudi	195	78.0
Non-Saudi	55	22.0
Working hours		
≤8	164	65.6
≥9	86	34.4

**Table 2:** Nurses’ perceptions of quality of nurse–physician communication in Emergency departments.

Item	Mean ± SD
Mutual Understanding	
Nurses’ difficulties in understanding what physicians mean [6]	2.83 ± 1.13
Physicians’ difficulties in understanding what nurses means [8]	3.20 ± 1.08
Openness	
Difficult or easy talking openly with physicians [1]	3.64 ± 1.04
Physicians listening to nurses [4]	346 ± 1.09
Receiving correct information or advice from physicians [7]	3.53 ± 0.99
Communication openness between nurses and physicians [9]	3.58 ± 1.01
Frustration with Interaction	
Feeling angry after interaction with physicians [12]	2.78 ± 1.11
Feeling frustrated after interaction with physicians [14]	2.66 ± 1.03
Feeling misunderstood after interaction with physicians [15]	2.84 ± 1.15
Feeling dissatisfied after interaction with physicians [17]	2.84 ± 1.07
Relevance and Satisfaction	
Feeling respected after interaction with physicians [18]	3.70 ± 0.99
Feeling pleased after interaction with physicians [16]	3.39 ± 0.98
Feeling satisfied after interaction with physicians [13]	3.51 ± 0.93
Level of understanding between nurses and physicians [11]	3.60 ± 0.94
The value of contact with physicians [10]	3.57 ± 0.95
Difficulty or easy to ask physicians for advice [2]	3.72 ± 0.96
Joyfulness of talking to physicians [5]	3.56 ± 1.04
Relevance of information provided by physicians [3]	3.46 ± 1.05
Total score	60.14

**Table 3:** Subscale scores of nurses’ perceptions of quality of nurse–physician communications in emergency departments.

Sub-Domain	Items	Range	Mean ± SD	Mean Score %
Relevance and satisfaction	8	8–40	28.55 ± 5.96	71.60
Openness	4	4–20	14.22 ± 3.33	71.65
Mutual understanding	2	2–10	6.03 ± 0.98	60.10
Frustration with interaction	4	4–20	11.13 ± 3.66	55.80

**Table 4:** Factors affecting nurses’ perceptions of quality of nurse–physician communication in emergency departments.

Factor	Group	*N*	Mean ± SD	Test Value	*p*-Value
Sex	Male	54	60.81 ± 9.94	t (0.797)	0.426
	Female	196	59.70 ± 8.75		
Age	30–35	77	57.33 ± 8.18	F (6.257)	0.002 *
	≥36	103	60.18 ± 9.55		
	<30	70	62.47 ± 8.40		
Level of education	Diploma	66	62.51 ± 8.93	F (4.200)	0.016 *
	Bachelor	147	58.70 ± 8.83		
	Master	37	60.29 ± 9.10		
Experience in ED (years)	1–5 years	77	57.98 ± 9.04	F (3.881)	0.022 *
	6–10	72	59.58 ± 9.36		
	>10	101	61.70 ± 8.47		
Job position	Bedside nurse	176	58.93 ± 9.44	F (3.999)	0.020 *
	Head nurse	40	61.87 ± 8.77		
	Nursing supervisor	34	62.94 ± 5.47		
Marital status	Single	75	58.01 ± 9.48	F (2.499)	0.084
	Married	166	60.76 ± 8.88		
	Divorced	9	61.00 ± 4.24		
Nationality	Saudi	195	59.42 ± 8.67	t (−1.732)	0.085
	Non-Saudi	55	61.80 ± 10.01		
Working hours	≤8	164	60.06 ± 8.05	t (0.273)	0.758
	≥9	86	59.73 ± 1065		

* Significant.

**Table 5:** Multiple linear regression of independent factors of nurses’ perceptions of quality of nurse–physician communication.

Factor	Unstandardized Coefficients	Coefficients Std Error	Standardized Coefficients	t	Sig.
(Constant)	57.180	2.368		24.144	0.000
Age	1.944	1.201	0.166	1.619	0.107
Level of education	−1.533	0.889	−0.108	−1.724	0.086
Experience in ED (years)	−0.037	1.074	−0.003	−0.035	0.972
job position	1.327	0.885	0.106	1.499	0.135

## Data Availability

The data presented in this study are available on request from the corresponding author.

## Supplementary Materials

The following supporting information can be downloaded at: https://www.mdpi.com/article/10.3390/healthcare11050645/s1, Supplementary S1: The questionnaire used in this study.
