# Supplementary material for: Nurses’ Perception Regarding the Quality of Communication between Nurses and Physicians in Emergency Departments in Saudi Arabia: A Cross Sectional Study
Source: Healthcare (Basel). 2023 Feb 22;11(5):645. doi: 10.3390/healthcare11050645 (PMC10000906; doi:10.3390/healthcare11050645)
Supplement: Supplementary file 1 [file healthcare-11-00645-s001.zip › healthcare-2186687-supplementary.pdf]

Supplementary S1: The questionnaire used in this study Part 1: Socio demographic data of healthcare providers

**1. Gender**	□ Male<br>□ Female
**2. Position.**	□ Nursing supervisor<br>□ Head-nurse<br>□ Beside nurse
**3. Education**	□ Diploma<br>□ Bachelor<br>□ Master<br>□ PhD
**4. Years of experience**	……….. Years
**5. Age**	……….. Years
**6. Marital status**	□ Married<br>□ Single<br>□ Divorced
**7. Nationality**	□ Saudi<br>□ Non-Saudi
**8. working hours per day**	□ ≤8<br>□ ≥9

Part (2): Nurse–physician communication

1. How difficult or easy do you find it to talk openly with the physicians working with this nursing home?

1	2	3	4	5
Very difficult				Very easy

2. How difficult or easy do you find it to ask physicians for advice?

1	2	3	4	5

Very difficult				Very easy

3. How often is the information or advice you get from physicians relevant?

1	2	3	4	5
Almost never				Always

4. How often would you say that physicians listen to what you have to say?

1	2	3	4	5
Almost never				Always

5. How often do you find it enjoyable to talk to physicians?

1	2	3	4	5
Almost never				Always

6. How often do you have difficulties understanding what physicians mean?

1	2	3	4	5
Almost never				Always

7. How often would you receive correct information or advice from physicians?

1	2	3	4	5
Almost never				Always

8. How often do have physicians have difficulties understanding what you mean?

1	2	3	4	5
Almost never				Always

9. How open is the communication between nurses and physicians in this nursing home?

1	2	3	4	5
Not at all				Extremely

10. How valuable do you find your contacts with physicians?

1	2	3	4	5
Not at all				Extremely

11. How much understanding is there between nurses and physicians in emergency?

1	2	3	4	5
None				A lot

12. How often do you feel angry after an interaction with a physician?

1	2	3	4	5
Never				Almost always

13. How often do you feel satisfied after an interaction with a physician?

1	2	3	4	5
Never				Almost always

14. How often do you feel frustrated after an interaction with a physician?

1	2	3	4	5
Never				Almost always

15. How often do you feel misunderstood after an interaction with a physician?

1	2	3	4	5
Never				Almost always

16. How often do you feel pleased after an interaction with a physician?

1	2	3	4	5
Never				Almost always

17. How often do you feel dissatisfied after an interaction with a physician?

1	2	3	4	5
Never				Almost always

18. How often do you feel respected after an interaction with a physician?

1	2	3	4	5
Never				Almost always
